# Improving the core functions of primary care in a Ugandan rural district

**DOI:** 10.4102/phcfm.v17i1.4782

**Published:** 2025-05-13

**Authors:** Innocent K. Besigye, Robert J. Mash

**Affiliations:** 1 Division of Family Medicine and Primary Care, Faculty of Medicine and Health Sciences, Stellenbosch University, Cape Town, South Africa; 2 Department of Family Medicine, Faculty of Medicine, Makerere University, Kampala, Uganda

**Keywords:** primary care, core functions, primary health care, co-design, intervention development

## Abstract

**Background:**

In many countries, the core functions of primary care (PC) continue to perform poorly and therefore need improvement, guided by interventions developed in collaboration with key stakeholders.

**Aim:**

This paper reports on the co-design of an intervention guided by the findings of the Primary Care Assessment Tool (PCAT) survey.

**Setting:**

The setting for the study was a rural Ugandan district.

**Methods:**

This was part of a multi-stage mixed methods study to evaluate the use of the PCAT in improving primary care performance. Key stakeholders in primary care system were purposively identified to participate in the design of the intervention. The intervention co-design involved presentation and discussion of the PCAT findings, two rounds of root cause analysis, selection of intervention focus area, design of the actual intervention and planning of implementation.

**Results:**

Ongoing care was selected for intervention among the poorly performing primary care core functions. Community members’ low awareness of the available services and low affiliation to their local primary health care (PHC) facility were identified as major contributors to the poor performance of ongoing care. Community dialogues as form of community engagement were selected as an intervention to improve the core primary care functions.

**Conclusion:**

The PCAT can generate findings to guide the development of interventions at the facility and district level to potentially improve the core functions of primary care.

**Contribution:**

A co-design process helped to navigate the pathway from the findings to the intervention design and its implementation strategy.

## Introduction

### Background

Since the declaration of Alma Ata in 1978, implementing primary health care (PHC) has remained on the global agenda as a key strategy for strengthening health systems.^1,2,3^ Primary health care is key to improving health status, while also reducing costs and improving equity.^3,4^ However, in many regions of the world, PHC remains weak amid increasing demands and complexity of health systems. The World Health Report 2008 described how primary care, the service delivery component of PHC should be more responsive to the needs of the people served and outlined the necessary reforms.^5^ Nevertheless, in 2018 a further global commitment was needed and expressed in the Astana Declaration.^2^

Strong PHC is key to achieving universal health coverage and the third sustainable development goal on health and wellbeing.^6^ In many low- and middle-income countries (LMICs), primary care continues to suffer from poor performance and inadequate funding despite being the level of care where most patients are seen and cared for.^7^ Primary health care shifts health systems towards more community-orientated, multisectoral and comprehensive primary care activities that address the majority of the population’s health needs.^8^

As countries continue to develop and strengthen their PHC systems, there is a need to measure performance, monitor improvement and inform strategies to improve performance. The World Health Organization (WHO) formulated a PHC measurement framework that includes the need to measure the core functions of primary care as part of service delivery.^9^ The core primary care functions include first contact access, continuity, coordination, comprehensiveness and person-centred care. The framework suggests the need to develop tools to support facility-level exit interviews that can measure these core functions.

Such functions can be measured by the Primary Care Assessment Tool (PCAT), which was originally developed at the Johns Hopkins Primary Care Policy Centre in the United States.^10^ The PCAT has been adapted for use in several African countries (South Africa, Malawi, Kenya and Uganda) and in other regions of the world.^11,12,13,14,15^ The Ugandan PCAT (UG PCAT) was used to measure the performance of the core functions of primary care together with its other domains in public primary care facilities in a rural district with the aim of identifying performance gaps for improvement.^16^

Most PCAT studies measure primary care performance but do not actively investigate how the findings can be used to improve performance at the facility and district level. The utility of the PCAT lies in its ability to guide interventions to improve performance at the micro-, meso- and macro-levels of the health system. The aim of this study was to co-design an intervention, with the local facility and district-level management, based on the findings of the UG PCAT and to demonstrate how the PCAT can enable strategies to improve primary care performance.

## Methods

### Study design

**FIGURE 1:**
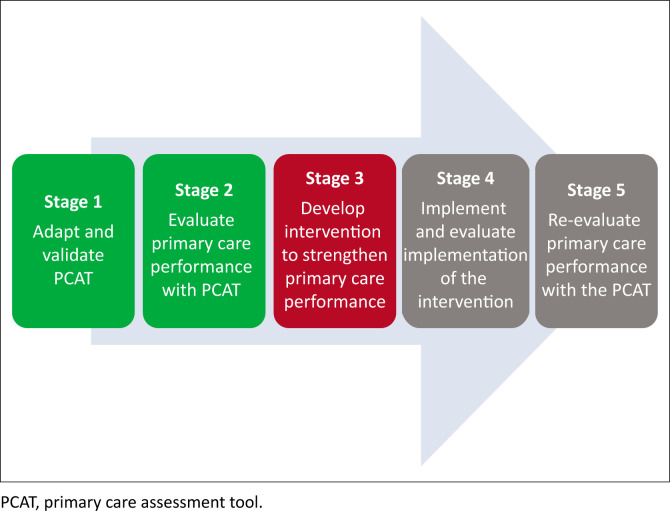
Multi-stage mixed methods study design.

This forms part of a multi-stage mixed methods study, as shown in Figure 1. The larger study used the locally validated PCAT (stage1) to evaluate the strength of primary care performance in Tororo district in terms of the core functions (stage 2). In this article, we report on stage 3, how these findings informed a co-design process with the district health services to design and develop an appropriate intervention that would strengthen primary care performance. Future work will assess implementation (stage 4) and the longer-term effects on performance (stage 5).

### Study setting

The study was located in Tororo district in Eastern Uganda on the Uganda-Kenya boarder. The district covers a total surface area of 1196 km^2^ with an estimated total population of 540 300 people. Most of the population is younger than 30 years with only primary school education and survives on subsistence farming.^17^ According to the annual health sector performance report 2020–2021, Tororo district remains among the poorest performing districts in Uganda in almost all health indicators.^18^

In Uganda, the District Health System (DHS), which is responsible for PHC, is under the stewardship of the local government. The DHS is composed of five levels of health facilities. Health Centre I (HCI) is a group of community members known as a Village Health Team (VHT) with no physical structure, whose main role is community mobilisation for health. Health Centre II (HCII) is a community dispensary offering ambulatory care, Health Centre III (HCIII) provides additional maternal and child health care services, Health Centre IV (HCIV) offers additional emergency obstetric care and the General Hospital (GH) at the apex also offers general medical and surgical services to inpatients. There is one GH, four health centre IVs, 18 health centre IIIs and 36 health centre IIs in Tororo District. The District Health Officer (DHO) leads the health district, assisted by a team of other managers. This District Health Team (DHT) is mandated to make decisions on the delivery of health services. The DHT plans health services and implements health policies. Nurses, clinical officers and non-specialist medical officers are the primary care providers. The DHT has powers to make binding decisions and plans for the district. The communities have a voice in the management structures through the Health Unit Management Committee (HUMC) that exists at every health facility.

### Selection of co-design team

The researcher purposively identified key stakeholders to ensure representation across all levels of primary care facilities within the district. The identified stakeholders included one member of the District Health Team (DHT), four health facility managers, four primary care providers and one HUMC member representing the community.

### Co-design process

The stakeholders were invited to a 4-day workshop that was facilitated by the author (IB). The workshop was held in the Family Medicine office at Tororo General Hospital. There were three specific objectives:

- To identify the strengths and weaknesses of current primary care performance and the underlying reasons for poor performance.
- To prioritise a specific focus for an intervention to improve performance.
- To design the intervention and prepare for implementation.

#### Presentation of results

The author (IB) presented the UG PCAT results and highlighted the key strengths and weaknesses in primary care performance. The details of the UG PCAT are described elsewhere.^14^

#### First round of root cause analysis

The stakeholders went through a brainstorming session^19^ to identify the core functions that were performing poorly and to generate insights into the possible reasons. The reasons for poor performance were categorised by the researcher under different PCAT domains and cross-checked with the participants. In some cases, the reasons were indirectly linked with the PCAT domains.

#### Prioritisation of focus area

Voting, based on a simple majority, selected one PCAT domain to focus on for improvement.

#### Second round of root cause analysis

Once the domain was selected, then further reasons for its poor performance were brainstormed.

#### Selection of focus area for the intervention

A nominal group technique (NGT)^20,21^ was then conducted to rank the reasons in terms of order of importance.

#### Brainstorming of possible interventions

The group brainstormed the different ways in which they could intervene with the top ranked reasons for poor performance.

#### Selection of intervention

Another NGT was done to rank the listed approaches in order to achieve consensus on what to include in the intervention. The highest-ranking approach was then focused on to design the intervention.

#### Design of intervention

A strengths-weaknesses-opportunities-threats (SWOT) analysis was done to consider the issues that might impact on implementation of the agreed intervention. The intervention needed to be feasible with the available resources and timeframe (over 6 months) as well as likely to make a difference. The final plan was developed into a logic model to identify the needed inputs, required activities and expected outputs and outcomes.

### Ethical considerations

This study was approved by the Health Research Ethics Committee at Stellenbosch (S20/04/103) and the Makerere University School of Medicine Research and Ethics Committee (# REC REF 2020-164). Written informed consent was sought from the participants before their participation in the study. The participants were informed that their participation was voluntary and that they can withdraw their participation at any stage of the process with no negative consequences. The study was carried out in accordance with relevant guidelines and regulations. As the study involved human subjects, their selection and participation were done in accordance with the Declaration of Helsinki.

## Results

Ongoing care, coordination of care, comprehensiveness of care and specifically the composition of the PHC team were identified as the poorly performing domains from the PCAT findings. Ongoing care was selected as the key domain to focus on for improvement. Reasons for poor performance in ongoing care are listed in Table 1 with their scores and ranking from the NGT. The reasons for poor performance in the other three domains are listed in Table 2.

**TABLE 1:** Reasons for poor primary care performance in ongoing care with the nominal group technique scores and ranking.

Reasons for poor performance in ongoing care	NGT score	Ranking
Community members are not aware of the services available at their nearest health facilities	7	1st
Weak community linkages. Although Village Health Team members are well connected in the community, they are volunteers, with low morale and motivation	6	2nd
Staff attitudes, for example, being rude to patients and inability to maintain confidentiality	4	3rd
No regular staff meetings to discuss service delivery. Facility meetings normally focus on welfare of staff and little or no focus on the users of the facilities	2	4th
Issues with opening hours, for example, staff not returning to work after lunch break and not working on weekends, particularly in lower-level facilities	1	5th
Erratic transfers of staff between health facilities affecting ongoing care	0	-
Health providers not creating relationships with patients	0	-
Health providers do not inform patients to come back for review	0	-

NGT, nominal group technique.

**TABLE 2:** Reasons for poor primary care performance in coordination of care, comprehensiveness and primary health care team composition.

Variable	Reasons for poor performance
Coordination of care	Health providers do not give adequate information to patients
	No feedback given on referrals to the referring facility
	Poor transfer of information (referral letters) when referring patients to higher PHC levels and sometimes just relying on the patients’ verbal explanation
Comprehensiveness of care	Regular stock-outs of essential medicines particularly in lower-level health facilities
PHC team	Human resource problems, for example, the district has only 64% of posts filled
	A lack of staff trained and competent in PHC to supervise activities of the district
Other important reasons	Community meetings that would ensure community involvement are not happening
	Unsupportive supervision from the district level with little or no focus on technical service delivery areas and the users of facilities. The focus is mainly on finances
	Community sensitisation and health education are no longer being done

PHC, primary health care.

A lack of awareness in the community on the availability of services and importance of ongoing care at the same facility were seen as the key issues to focus on in designing an intervention. This was referred to as a ‘lack of community sensitisation’. Table 3 shows the different approaches to community sensitisation that were suggested and how these were ranked. The approaches were community dialogues, health education, refocusing health facilities towards users instead of staff welfare, facility-level sensitisation of staff, use of media and signage in the facilities.

**TABLE 3:** Suggested approaches to community sensitisation and their nominal group technique scores and ranking.

Suggested method of sensitisation	NGT score	Ranking
Community dialogues to discuss with community members the importance of ongoing care and informing them of the available services at the health facilities	7	1st
Health education about the importance of seeking care from the same health facility	7	1st
Facility meetings focusing on the welfare and better experience of users of health facilities	3	2nd
Facility-level sensitisation of staff by the District Health Team and facility managers to improve staff attitudes and be more person-centred	2	3rd
Use of media (e.g. radio talk shows) to inform the public about the services available at the health facilities	1	4th
Use of signage at the entrance of health facilities to show the services available	0	-

NGT, nominal group technique.

Community dialogues and health education had the same rankings. The group then discussed and unanimously agreed that the intervention should focus on community dialogues as this will also encompass health education. The final intervention agreed upon was: ‘Use of community dialogues for improving on-going care in primary care’.

The stakeholder group decided to implement the intervention in one health sub-district (Mukuiju) that contained one HCIV, nine HCIIIs and six HCIIs. The key idea was for the team that conducts outreach into the community for immunisations to also conduct community dialogues. The project’s core team was purposively selected according to their roles at the health facilities and their involvement in outreach. Two nurses at each facility, the family planning focal person and the person in charge of immunisations, were selected because of their active roles in outreach as well as two other facility-based staff to conduct community dialogues at the health facilities.

The project intended to make community engagement part of the daily health facility activities and to make use of an existing community-based service to conduct the dialogues. The intention was to sensitise community members on the available services and the importance of seeking care from the same health facility, particularly the closest one. The VHT members would be requested to mobilise community members within their catchment areas to participate in community dialogues. Health providers going out for immunisation outreaches in the communities would conduct the community dialogues with the help of a community dialogue guide.

**FIGURE 2:**
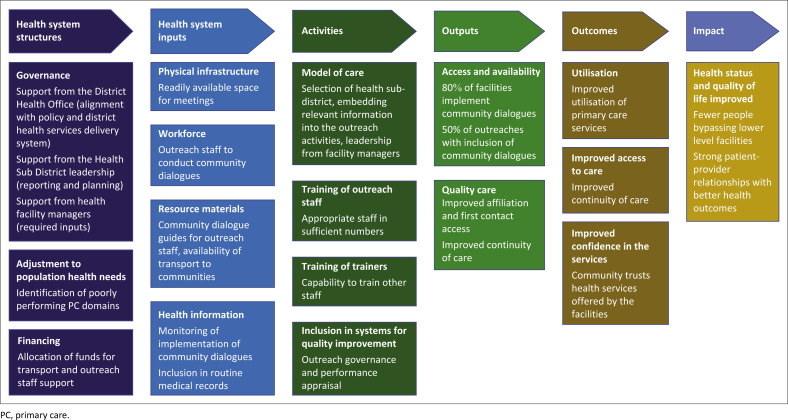
Logic model to guide intervention implementation.

The group’s SWOT analysis of the proposed intervention is shown in Table 4 and the final logic model to guide implementation in Figure 2.

**TABLE 4:** A strengths-weaknesses-opportunities-threats analysis of the intervention implementation.

**Strengths** Availability of staff with experience in health education Availability of transport to the community outreach sites Supportive administration within the health facilities	**Weaknesses** Poor packaging of information with different staff relaying different information Lack of commitment from the VHTs given that their participation is voluntary Low staff numbers and high patient numbers
**Opportunities** Availability of already established facility and community governance structures that are used in the delivery of health services Routinely conducted outreach activities Support from the district health office Communities are usually very receptive to health interventions	**Threats** Low turnout of community members Unfavourable weather conditions (rainy or hot weather) given that outreach activities are conducted outdoors Negative perceptions from community members towards the intervention Political interference with some local politicians passing on different messages

VHT, Village Health Team.

### Development of the community dialogue guide

A guide for use by the implementation team was developed by the researcher, discussed and agreed upon with the stakeholders. The implementation team was trained on using the guide during community dialogues.

## Discussion

### Summary of key findings

The co-design process successfully developed an intervention that was based on the findings of the UG PCAT and the core functions of primary care. The facility and district-level stakeholders were able to make sense of the findings and identified ongoing care, coordination of care and comprehensiveness of care as the core functions that needed strengthening. They were able to explore the root causes of poor performance and prioritised low community awareness of the services available and the importance of ongoing care as the key issues to focus on. They were able to identify community dialogues with health education as the most feasible intervention. Finally, they were able to design the intervention and plan an implementation strategy. This process demonstrates the ability of the PCAT findings to support district-level development of interventions to improve PHC.

### Discussion of key findings

The key stakeholders for PHC services provision in the district co-designed the intervention with the researcher (IB). Using this low level of community engagement,^22^ that involved providing information to the stakeholders to only understand the issues concerning the performance of primary care may have undermined the available community resources that could have assisted with implementing the intervention.

Although the stakeholders identified ongoing or continuity of care as the key domain to focus on, the intervention was actually more about improving affiliation to a specific PHC facility and utilisation of services. In terms of the core functions of primary care, this is more targeting first contact access. While improving first contact access can be seen as a prerequisite for improving continuity of care, it may also indicate a lack of familiarity with the concepts included in the core primary care functions. Members of the DHT and primary care providers had difficulty understanding the concepts of core functions of primary care. Continuity of care is usually more concerned with the consistent availability of the medical record (informational continuity) and the quality of the relationship with a particular provider (relational continuity).^23^ This lack of understanding may also reflect that health systems and management teams tend to focus on disease-orientated programmes rather than cross-cutting functions of primary care.

Community engagement can allow design of interventions that are contextually and culturally sensitive, which promotes acceptability.^24^ Effective community engagement requires locally generated evidence, easily understood by the representative group of community stakeholders. In this intervention design, the PCAT results from the study district guided the intervention design, and the intervention has the potential to improve the quality of primary care services. However, the co-design team only included one community representative, and effective community engagement would require more time and energy to achieve a shared understanding of the results and common ground during intervention design.

On the one hand, it seems quite progressive that the stakeholders opted for community dialogues. On the other hand, one wonders whether this was not a way of avoiding pointing a finger at themselves in terms of what needs to be fixed. The stakeholders who were mainly health managers and providers appeared to be saying that the community was the main problem and therefore needed to be educated. They seem to have ignored or avoided dealing with some serious issues in their own services with staff meetings, attitudes and accountability. More extensive community engagement and empowerment in the process could have led to a different set of priorities.

Although the community dialogues were intended to motivate and share information with the community, they are likely to solicit feedback from the community on their own experiences and perspectives with regard to the quality of services provided. A key issue in evaluating implementation will be how such feedback is received and responded to. It should also be noted that the stakeholders opted for only one activity, yet literature stipulates that multi-faceted approaches are likely to be more successful in improving the quality of services.^25^ Therefore, an intervention that focuses on both the community and the health services may have been more appropriate.

Community dialogues as an approach to community engagement have been used to improve the health of populations and communities as well as identifying health concerns within communities.^26,27^ Community dialogues are premised on the Integrated Model of Communication for Social Change.^28^ This model presupposes that a stimulus is necessary to trigger a dialogue within community members about problems existing within the community. It is a dynamic and iterative process at individual and community level resulting in social change. In this intervention, community dialogues will involve staff from health facilities interfacing with members of the communities served. A group of 20–30 community members is required for a successful community dialogue. Community dialogues involve a level of listening to the community with a goal of empowering the community through participation and collaboration as opposed to just ‘informing’ or educating people, a focus of most community engagements.

The implementation of this intervention was deliberately embedded within the already existing facility and community activities and structures. This will ensure that no direct incremental costs are required to implement or sustain the initiative. However, the health providers implementing the intervention will incur indirect opportunity costs in terms of the extra time and effort required, amid existing challenges of low human resources for health and large patient numbers. Managers will need to approve the opportunity costs, and healthcare workers will need motivation to ensure successful implementation. This implies that they see it as important and are confident in their ability to deliver the intervention.

This implementation strategy was informed by literature that describes community engagement approaches to require substantial financial and human resources as well as time.^29^ Therefore, only one health sub-district was selected for the intervention with the possibility of scale up in the future based on the evaluation results. The implementation of the intervention is ongoing and will be evaluated for its acceptability, appropriateness, adoption, feasibility, fidelity, coverage, cost and sustainability.

### Strengths and limitations

A systematic approach guided by the UG PCAT findings was used to co-design an intervention together with the stakeholders who also participated in the planning of its implementation. All the key stakeholders were involved to ensure that all the perspectives were considered. Generally, the approach used was collaborative and participatory. Known open-ended approaches of information generation (e.g. brainstorming, root cause analysis) and consensus building (e.g. NGT) were used. This approach ensured ownership of the intervention by the stakeholders.

A SWOT analysis was done to help in understanding both the internal and external environments within which the intervention will be implemented. This is presumed to allow leveraging on the strengths and opportunities to improve the success of the intervention while mitigating the weaknesses and threats.

The stakeholders involved in the co-design process had minimal inclusion of community members. Therefore, it is not clear whether their active participation and more balanced power among stakeholders would have led to different priorities in improving PHC. This should be tested with a more balanced group of stakeholders in the future.

### Implications

The PCAT findings can be used to guide development of interventions at facility and district levels for quality improvement in the core functions of primary care for better quality services. This is a different approach to the more common and historical disease-orientated approach to quality improvement.^30,31,32^ However, health care managers and leaders may need more training in the core functions of primary care to fully understand these concepts and their importance for service delivery.

The co-design process was successful in developing an intervention that was implemented in the district and seen as acceptable and appropriate. However, future co-design processes should have more community participation to ensure that the community voice also guides the prioritisation, root cause analysis and design steps.

Further research will evaluate the implementation of community dialogues and whether this leads to any measurable change in the core functions of primary care.

## Conclusion

The PCAT can guide development of interventions to improve the core functions of primary care services at the district and facility levels. A co-design process successfully navigated the pathway from PCAT findings to design of an intervention and the implementation strategy. The process could have benefited from more community participation. Health service managers and leaders also needed more familiarity with the key concepts embedded in the core functions. Further research will evaluate implementation and the effect on the core primary care functions.

## References

[1] World Health Organization. Declaration of alma-ata. Copenhagen: Regional Office for Europe; 1978.

[2] World Health Organization. Declaration of Astana: Global Conference on Primary Health Care: Astana, Kazakhstan, 25 and 26 October 2018. Geneva: World Health Organization; 2019.

[3] World Health Organization. Primary health care [homepage on the Internet]. Geneva: WHO; 2023 [cited 2023 Dec 01]. Available from: https://www.who.int/news-room/fact-sheets/detail/primary-health-care#:~:text=PHC%20is%20the%20most%20inclusive,as%20the%20COVID%2D19%20pandemic

[4] Macinko J, Starfield B, Shi L. The contribution of primary care systems to health outcomes within Organization for Economic Cooperation and Development (OECD) countries, 1970–1998. Health Serv Res. 2003;38(3):831–865. 10.1111/1475-6773.00149 12822915 PMC1360919

[5] Van Lerberghe W. The world health report 2008: Primary health care: Now more than ever. Geneva: World Health Organization; 2008.

[6] World Health Organization. A vision for primary health care in the 21st century: Towards universal health coverage and the Sustainable Development Goals. Geneva: World Health Organization; 2018.

[7] Primary health performance initiative. A Message from the Steering Committee of the Primary Health Care Performance Initiative (PHCPI) [homepage on the Internet]. PHCPI; 2022 [cited 2023 Aug 24]. Available from: https://www.improvingphc.org/transition

[8] World Health Organization. Operational framework for primary health care: Transforming vision into action. Geneva: World Health Organization; 2020.

[9] World Health Organization. Primary health care measurement framework and indicators: Monitoring health systems through a primary health care lens. Web annex: Technical specifications. Geneva: World Health Organization; 2022. Report No.: 924004423X.

[10] Shi L, Starfield B, Xu J. Validating the adult primary care assessment tool. J Fam Pract. 2001;50(2):161–175. 10.1037/t77102-000

[11] Manga N, Sayed A-R, Bhagwan S, Bresick G, Le Grange C. Adaptation and cross-cultural validation of the United States Primary Care Assessment Tool (expanded version) for use in South Africa. Afr J Prim Health Care Fam Med. 2015;7(1):1–11. 10.4102/phcfm.v7i1.783 PMC4656921 26245610

[12] Dullie L, Meland E, Hetlevik Ø, Mildestvedt T, Gjesdal S. Development and validation of a Malawian version of the primary care assessment tool. BMC Fam Pract. 2018;19:1–11. 10.1186/s12875-018-0763-0 29769022 PMC5956555

[13] Mohamoud G, Mash R. The quality of primary care performance in private sector facilities in Nairobi, Kenya: A cross-sectional descriptive survey. BMC Prim Care. 2022;23(1):120. 10.1186/s12875-022-01700-3 35585488 PMC9114290

[14] Besigye IK, Mash R. Adaptation and validation of the Ugandan Primary Care Assessment Tool. Afr J Prim Health Care Fam Med. 2023;15(1):3835. 10.4102/phcfm.v15i1.3835 36744453 PMC9900308

[15] Fracolli LA, Gomes MFP, Nabão FRZ, Santos MS, Cappellini VK, Almeida ACCd. Primary health care assessment tools: A literature review and metasynthesis. Cien Saude Colet. 2014;19:4851–4860. 10.1590/1413-812320141912.00572014 25388193

[16] Besigye IK, Mash RJ. Primary care performance in a Ugandan rural district: cross-sectional descriptive study. BJGP Open. 2024;BJGPO.2024.0105. 10.3399/BJGPO.2024.0105 PMC12421270 39505399

[17] Uganda Bureau of Statistics. Statistical abstract 2021 [homepage on the Internet]. UBOS; 2021 [cited 2023 Dec 01]. Available from: http://library.health.go.ug/sites/default/files/resources/UBOS%20Statistical%20Abstract%202021.pdf

[18] Ministry of Health. Annual health sector performance report 2020/21 [homepage on the Internet]. Kampala: MOH; 2021 [cited 2023 Dec 01]. Available from: http://library.health.go.ug/sites/default/files/resources/Annual%20Health%20Sector%20Performance%20Report%202020-21-1.pdf

[19] Hidayanti WI, Rochintaniawati D, Agustin RR. The effect of brainstorming on students’ creative thinking skill in learning nutrition. J Sci Learn. 2018;1(2):44–48. 10.17509/jsl.v1i2.8738

[20] Centre for Disease Control. Ganining consensus among stakeholders through the nominal group technique [homepage on the Internet]. CDC; 2018 [cited 2023 Aug 28]. Available from: https://www.cdc.gov/healthyyouth/evaluation/pdf/brief7.pdf

[21] Harvey N, Holmes CA. Nominal group technique: An effective method for obtaining group consensus. Int J Nurs Pract. 2012;18(2):188–194. 10.1111/j.1440-172X.2012.02017.x 22435983

[22] The open university. Levels of community engagement [homepage on the Internet]. 2016 [cited 2024 Mar 08]. Available from: https://www.open.edu/openlearncreate/mod/oucontent/view.php?id=80596&section=6

[23] Haggerty JL, Reid RJ, Freeman GK, Starfield BH, Adair CE, McKendry R. Continuity of care: A multidisciplinary review. BMJ. 2003;327(7425):1219–1221. 10.1136/bmj.327.7425.1219 14630762 PMC274066

[24] Erku D, Khatri R, Endalamaw A, et al. Community engagement initiatives in primary health care to achieve universal health coverage: A realist synthesis of scoping review. PLoS One. 2023;18(5):e0285222. 10.1371/journal.pone.0285222 37134102 PMC10156058

[25] Hulscher M, Wensing M, Grol R. Multifaceted strategies for improvement. Improving patient care: The implementation of change in health care. New Jersey: Wiley-Blackwell; 2013; p. 278–288.

[26] Consortium M. Community dialogues for healthy children [homepage on the Internet]. London; 2012 [cited 2024 Jan 02]. Available from: https://www.malariaconsortium.org/media-download-file/201305101032/-/learning_paper_community_dialogue_oct_2012.pdf

[27] Najjuma SM, Kyaddondo D. Exploring ‘spaces for community dialogue’ among adults and children in collective identification, sharing and mitigation of HIV/AIDS concerns in Uganda. J Glob Health Rep. 2023;7:e2023008. 10.29392/001c.74381

[28] Figueroa MEK, Rani DL, Manju Lewisnline G. Communication for social change: An integrated model for measuring the process and its outcomes. New York: The Rockefeller Foundation; 2003.

[29] Skivington K, Matthews L, Simpson SA, et al. A new framework for developing and evaluating complex interventions: Update of Medical Research Council guidance. BMJ. The Rockefeller Foundation: New York; 2021;374. 10.1136/bmj.n2061 PMC8482308 34593508

[30] Cairncross S, Periès H, Cutts F. Vertical health programmes. Lancet. 1997;349:S20–S21. 10.1016/S0140-6736(97)90079-9

[31] Onwe FI, Okedo-Alex IN, Akamike IC, Igwe-Okomiso DO. Vertical disease programs and their effect on integrated disease surveillance and response: Perspectives of epidemiologists and surveillance officers in Nigeria. Trop Dis Travel Med Vaccines. 2021;7(1):28. 10.1186/s40794-021-00152-4 34593034 PMC8483794

[32] Mthembu Z, Mogaka JJO, Chimbari MJ. Community engagement processes in low- and middle-income countries health research settings: A systematic review of the literature. BMC Health Serv Res. 2023;23(1):457. 10.1186/s12913-023-09466-9 37158864 PMC10169489

